# Nevirapine (NVP) vs ritonavir-boosted atazanavir (ATV/r) combined with tenofovir/emtricitabine (TDF/FTC) in first-line therapy: NEWART 48-week data

**DOI:** 10.1186/1758-2652-13-S4-P4

**Published:** 2010-11-08

**Authors:** E DeJesus, A Mills, L Bhatti, C Conner, S Storfer

**Affiliations:** 1 Orlando Immunology Center, Orlando, FL, USA; 2 Anthony Mills MD Inc., Los Angeles, CA, USA; 3 AIDS Healthcare Foundation, Los Angeles, CA, USA; 4 Boehringer Ingelheim Pharmaceuticals Inc., 900 Ridgebury Rd., Ridgefield, CT, USA

## Purpose of study

Until recently, few prospective data existed to indicate safety/efficacy of NVP with entry CD4 count restricted to <250 (women) or <400 (men) cells/mm^3^. Two phase IV trials, ARTEN and NEWART, compared virologic safety/efficacy of NVP vs ATV/r on a background of TDF/FTC in HIV+ patients within these CD4 ranges. NEWART (US) was designed to be confirmatory of ARTEN and to supplement ARTEN data [1].

## Methods

In NEWART, treatment naïve patients were randomized to open-label NVP 200mg BID (lead-in dose 200mg QD x 14 days) or ATV/r (300/100mg) plus TDF/FTC (300/200mg). Women had CD4 counts (cells/mm3) <250; men <400. Primary endpoint was virologic response prior to and at week 48 defined as confirmed HIV viral load (VL) <50 copies/mL without subsequent rebound or therapy change. A point estimate of -6.5% or higher for difference in proportion of responders (NVP-ATV/r) was considered consistent with a successful ARTEN study.

## Summary of results

152 patients were treated: 89% male, 68% White, 31% Black. At baseline, mean log10 VL was 4.9 and median CD4 count (cells/mm3) was 176 (NVP) and 193 (ATV/r). Table 1 summarizes key outcome measures. Mean plasma lipid (mg/dL) changes from baseline through 48 weeks (last observation carried forward) were as follows (NVP and ATV/r arms, respectively): total cholesterol (TC) 18.2 and 13.8 (P=0.73); HDL cholesterol (HDLc) 9.6 and 3.5 (P=0.016); LDL cholesterol (LDLc) 8.7 and 6.9 (P=0.93); and triglycerides -4.7 and 8.4 (P=0.36).

**Table 1:** 

Outcome Measure (48 wks)	NVP 200 mg BID (n=75)	ATV/r 300/100 QD (n=77)	P value	Difference (95% CI) from model adjusting for screening VL and CD4+
Virologic Response (VR)	46 (61.3%)	50 (64.9%)	0.71	-4.1% (-18.3% to 10.1%)

Virologic Failure (VF), Protocol Defined	10 (13.3%)	12 (15.6%)	0.63	-2.6% (-13.1% to 7.9%)

Early withdrawals†	24	18		

- due to investigator-defined VF	5	0		

- due to adverse events	9*	9		

- due to other reason	10	9		

Mean Change in TC/HDLc [baseline to week 48 (LOCF)]	-0.38	-0.02	0.038	-0.33 (-0.64 to -0.02)

*NVP arm: 1 DAIDS grade 3 hepatobiliary AE (viral hepatitis); no grade 3/4 rash †2 deaths: 1-NVP (suicide); 1-ATV/r (lymphoma)

For NEWART and ARTEN (combined), VR for NVP 200mg BID was 65% (171/263) vs 65% for ATV/r (176/270) [P=0.75 (CI= -7.7% to 10.7%)].

## Conclusions

NVP + TDF/FTC was noninferior to ATV/r + TDF/FTC. Although trial discontinuations were greater in the NVP arm, VR was similar because of less documented VF. HDLc increased and TC/HDLc decreased significantly more for NVP than for ATV/r.
